# Association between the starting age of non-parental Early Childhood Education and Care (ECEC), and psycho-social problems in adolescence in West and East Germany – a natural experiment using data from the German Health Interview and Examination Survey for Children and Adolescents (KiGGS)

**DOI:** 10.1186/s40359-023-01447-1

**Published:** 2023-11-20

**Authors:** Ying Zhou, Annelene Wengler, Gabriele Doblhammer

**Affiliations:** 1 Institute for Sociology and Demography, University of Rostock, Rostock, Germany; 2 Department of Epidemiology and Health Monitoring, Robert Koch Institute, Berlin, Germany; 3 German Center for Neurodegenerative Disease, Bonn, Germany

**Keywords:** Early childhood education and care (ECEC), ECEC-start-age, Psycho-social problems

## Abstract

**Introduction:**

The study aimed to investigate the association between the start age of non-parental Early Childhood Education and Care (ECEC) and psycho-social problems in adolescence. The similarities and differences between West and East Germany were also investigated in a natural experiment.

**Methods:**

Our sample consisted of 1022 children (621 from West Germany, 401 from East Germany) aged 3–4 years at wave 2003–2006 that were followed up to wave 2014–2017 as adolescents (mean ± SD age = 14.4 ± 0.03 years) in the KiGGS study. The psycho-social problems were measured by the parent-reported Strengths and Difficulties Questionnaire (SDQ) at wave 2014–2017. Linear regression was used to explore the relationship between ECEC-start-age and psycho-social problems in adolescence in Germany, and stratified by West and East Germany.

**Results:**

Those who started ECEC between 2 and 3 years old (reference) had the lowest scores of psycho-social problems in the whole Germany and in West Germany in adolescence. In comparison, those who started ECEC older than 3 years old had higher scores of internalizing psycho-social problems in both West Germany (with statistically significant results) and East Germany (with a relatively larger effect size but insignificant results). Those who started ECEC younger than 1 year old had statistically significant higher scores for externalizing psycho-social problems in West Germany, even though less children started ECEC younger than 1 in West Germany compared to East Germany. This significant association was not found in East Germany. Those who started ECEC between 1 and 2 years old tended to have higher scores of externalizing psycho-social problems in both West and East Germany.

**Conclusion:**

The results suggest that if children start ECEC older than 3 years or younger than 2 years, more attention needs to be given to internalizing or externalizing psycho-social problems respectively. The regional differences for children younger than 1 year old may suggest a selection effect in West Germany where only fewer parents bring babies to ECEC, while the regional similarities for children over 3 years old indicate the importance of providing access to ECEC for children over 3 years old.

**Supplementary Information:**

The online version contains supplementary material available at 10.1186/s40359-023-01447-1.

## Background

The request for and use of non-parental Early Childhood Education and Care (ECEC) at a young age has increased in Germany and other high- and middle-income countries in recent years [1]. ECEC refers to any regulated non-parental arrangement that provides education and care for children from birth to compulsory primary school age [2]. It includes center-based ECEC and family-based non-parental daycare. In Germany, the use of non-parental ECEC for children under 3 years of age increased remarkably between 2002 and 2019 from 9 to 34%; and for children over 3 years of age from 90 to 93% [3, 4].

Germany was divided into German Democratic Republic (GDR) and Federal Republic of Germany (FRG) from 1949 to 1990. The different historical background resulted in distinct cultural and societal norms regarding women’s employment and family roles which continues to today. In GDR women were encouraged to work by emphasizing the principle of equal pay for equal work, providing a generous maternity leave policy, and available free non-parental ECEC. Housewives were devalued by being described as “parasites” in an environment where soviet-ordained gender equality was popular. On the other hand, in FRG women were discouraged to work because of tax and benefit system for dual-earner families and scarce non-parental ECEC. Working mothers were devalued and described as “raven mothers” in an environment where more traditional gender-role attitude were apparent [5]. Therefore, regional difference between West and East Germany affects the proportions of women in employment (56% women in West Germany and 89% women in East Germany worked in 1989; 12% mothers in West Germany and 30% mothers in East Germany worked full-time in 2017), and for the amount of ECEC available and its use (8% of children 0–3 year old in West Germany and 40% of 0–3 year old children in East Germany attended ECEC in 2006) [3, 5, 6]. The cultural difference regarding the role of women still exists today, several decades after the reunification [5]. Mothers in East Germany still behave according to the more egalitarian gender norms in which they grew up, and return back to work earlier and tend to work more hours after childbirth than mothers in West Germany [5]. However, with reunification, the GDR adopted the political, economic and legal institutions of West Germany since 1990. This included 1 year paid parental leave from 2005 with 36 months of parental leave in total available per child. The different development in the two parts of Germany provides an opportunity to study the effect of ECEC on psycho-social problems in adolescence as a sort of *natural experiment*, which, similarly to twin studies, can control for unmeasured or unknown confounders to some extent.

Psycho-social problems are major health problems with a prevalence of around 17 − 20% in children and adolescents in Germany [7]. They have a significant impact on their performance potential, quality of life, social participation, and lead to significant medical costs for society [8]. Psycho-social problems can be differentiated as externalizing and internalizing problems. Externalizing problems includes aggressive and oppositional behavior, high impulsivity and hyperactivity; Internalizing problems includes high social anxiety, depressive symptoms and withdrawal [8].

So far, the association between ECEC attendance and children’s psycho-social development is inconclusive. There is evidence of the benefits of ECEC on social and emotional functioning [2, 9] and, conversely, evidence of negative outcomes of ECEC, including insecure attachment, behavioral disorders and aggression [10–12]. In addition, some studies showed few or missing effects of ECEC [13, 14]. These inconsistencies of the findings may be due to the differences in the age at which ECEC started, as well as the intensity and quality of ECEC [2, 15].

Particular attention should be paid to the age at which ECEC begins. Previous studies reported early ECEC as a risk factor for insecure attachment and externalizing psycho-social problems, especially in the case of extensive, unstable, or low quality of ECEC exposure [10, 16]. However, other studies showed that the early childhood years are a sensitive period for social and language development [16]. Attending ECEC early is likely to provide more opportunities to play and interact with peers, to acquire social, language skills and to have close friends, which can reduce the risk of internalizing problems [17].

In addition, it remains questionable whether and to what extent the influence of ECEC on psycho-social problems continues beyond early childhood. Most studies investigating the effects of ECEC on psycho-social problems had a short-term follow-up, such as up to primary school [1, 2]; or are limited to investigating long-term effects on academic achievements or on secure attachment states of mind only [18, 19]. Among the few studies investigating the long-term effects of ECEC on psycho-social problems, an American study has reported that greater exposure to ECEC from birth to 4.5 years predicted more teacher-reported externalizing problems at age 12 [20].

Furthermore, in the literature there are also concerns about the internal and external validity of the studies regarding child-care and children’s psycho-social problems. One major concern regarding the internal validity is the potential selection bias, which is difficult to well control by using conventional statistical approaches such as covariate-adjusted correlations in observational studies [21]. For example, parental and family interaction outside ECEC, as an important factor for psycho-social development [22], is difficult to be fully controlled for by using conventional statistical approaches in the analysis of the association between ECEC and children’s psycho-social problems. Another critical limitation of previous studies is the heavy reliance on samples from the United States of America, which has limited the external validity [21]. Yet, in recent years there has been a growing body of research in this area in Europe [13].

Data from the KiGGS study (German Health Interview and Examination Survey for Children and Adolescents) allows us to investigate the long-term association between ECEC-start-age and psycho-social problems in adolescence, and its similarities and differences between West and East Germany in a natural experiment. Our study can contribute to this field of research, in particular regarding the following three points: First, we assess psycho-social development long time after ECEC started. Second, the natural experiment comparing the regional differences and similarities between West and East Germany may help control some unmeasured or unobserved confounders, thus reducing the selection bias and improving the internal validity. Third, our study attempts to provide more socio-political variations by using German samples and by even categorizing the samples into West and East Germany, to contribute to the external validity in this area of research.

## Methods

### Data source

We used data from the KiGGS study, a prospective cohort study [23, 24] of 17,640 children who were first interviewed in 2003–2006 (baseline wave) and followed up in two waves until 2014–2017 (wave 2). Since telephone interviews were used in wave 1, whereas self-administered questionnaires were used in the baseline wave and wave 2, wave 1 was excluded from our analysis. Wave 1 data were only used for the classification of the age at which ECEC started, because this age could only be clearly defined by using the data from baseline wave and wave 1 together (described below).

### Analysis sample

**Fig. 1:**
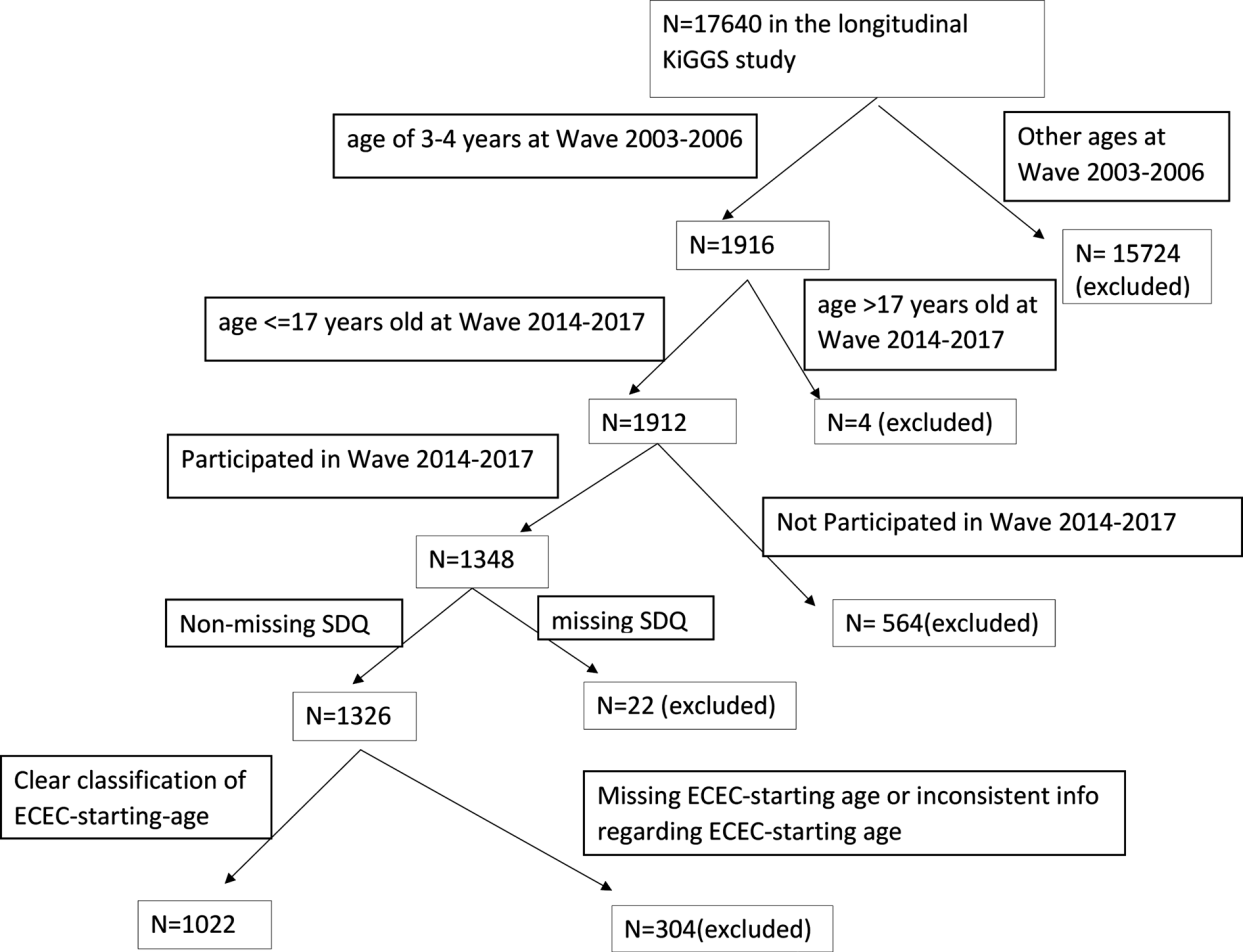
Flow chart for the sample

Our analysis sample included children who were 3 and 4 years old at the baseline wave and those who were younger than 18 years old and participated in wave 2. In addition, only children with complete SDQ (Strengths and Difficulties Questionnaire) and ECEC-start-age were included, resulting in an analysis sample of 1022 children (Fig. 1).

We selected children ≥ 3 years old at the baseline wave to ensure a clear classification of ECEC-start-age based on the data from the baseline wave and wave 1. We excluded children aged 5 and older, as they may be preparing to start school or may even be in school in Germany.

### Instruments

#### Psycho-social problems

We used the total difficulties score and four subscales of the SDQ at wave 2 to measure psycho-social problems in adolescence [25]. The score for externalizing psycho-social problems was obtained by summing up the scores for conduct problems and hyperactivity. The score for internalizing psycho-social problems was obtained by summing up the scores for emotional symptoms and peer problems. Each score (for externalizing or internalizing psycho-social problems) ranged from 0 to 20. The sum of the scores for externalizing and internalizing psycho-social problems made up the total score of SDQ, which accordingly ranged from 0 to 40. The higher the scores are, the larger psycho-social problems the children may have.

#### ECEC-start-age

The age at which ECEC started (ECEC-start-age) was derived from the data of the baseline wave and wave 1, based on the following two questions: “Was or is your child only cared for in the family before starting school?”. If the answer was no, then parents were asked about the ECEC-start-age. The specific rules for deriving the ECEC-start-age can be found in Supplementary Material 1.

#### Potential confounders

All potential confounding variables – SES (Socioeconomic Status), migration status, number of older siblings (order of birth), family situation and family cohesion, birth weight, obesity/overweight, maternal age at birth, maternal employment status and region (West/East Germany) - were collected at the baseline wave, except for the age, type of schooling, and parental divorce, which were collected or derived at wave 2. Exact operationalizations and some previous findings on these confounders can be found in the Supplementary Materials 2 and 3.

### Statistical analysis

We described the characteristics of the sample by using means and standard deviations for continuous variables and percentages for categorical variables for Germany as a whole, and by region (i.e. West or East Germany). In the bar charts, the mean values and 95% confidence intervals (CIs) of psycho-social problems in adolescence were presented by ECEC-start-age group. In an additional analysis, we used the Chi-square tests to analyze the distribution of SES and other baseline variables of interest by ECEC-start-age in West and East Germany separately. To ensure the representation of data at a national level, a cross-sectional weighting factor was applied to the baseline data, which was calculated to correct for deviations between the net sample (baseline data) and the population structure (as of 31 December 2004). The weighing factor is based on age, gender, region (West or East Germany) and nationality [23]. A longitudinal weight was applied to the wave 2 data to compensate for possible sample bias due to selective re-participation in the follow-up study [26].

We used linear regression to explore the relationship between ECEC-start-age and psycho-social problems in adolescence in the whole Germany, controlling for age, gender and other potential confounders as mentioned above. We also ran a similar linear regression, but stratified by West and East Germany. In an additional analysis, we added an interaction variable of region (West/East Germany) and ECEC-start-age in the linear regression of the whole Germany. In all linear regressions, we used the longitudinal weight, and the category “Age 2–3” as the reference group for the ECEC-start-age. Furthermore, linear regression assumptions, including normality and homoscedasticity, were checked using standardized residual graphics including histograms, normal quantile-quantile (QQ) plots and scatterplots of standardized residuals versus predicted values. We used the “PROC SURVEYREG” procedure in the SAS for a robust variance estimation method (Taylor series linearization method) to address the deviation from linear regression assumptions. A sensitivity analysis was performed to compare the results with and without outliers, which were defined as values more than 2 standardized residuals away from zero in the linear regression. All analyses were performed in SAS 9.4 (SAS Institute, Cary NC).

## Results

The children in our sample were on average 14.4 (± 0.03) years old at wave 2 (see Table 1). The sample was almost equally divided between girls and boys; 41.1% of our sample started ECEC at the age of 2–3 years, followed by 22.4% who started ECEC older than 3 years but before starting school. All other ECEC-age-groups accounted for about 10% of the children. At baseline wave 57.3% of the families belonged to the middle SES group, 16.5% to the low SES group and 25.2% to the high SES group; 16.0% of the children had a migrant background. The children were mostly (37.3%) the second child in the household and at baseline wave, 86.3% of the children lived with their natural parents in the same household. 16.7% of the parents were divorced by wave 2. Based on the data collected at baseline wave, most children were born with a birth weight of 2500-4000 g (78.8%) and were neither obese nor overweight (89.5%) when they were 3–4 years old. Most mothers gave birth at the age of 25–34 years (60.7%) and were unemployed (including housewife, or on parental leave etc.) (47.7%) when their child was 3–4 years old.

**Table 1:** Descriptive data for sample regarding baseline characters, and other potential confounders^a^

Character/Confounder	Category	Mean (Standard Deviation) / Number (%)		
		Total (n = 1022)	West Germany (n = 621)	East Germany (n = 401)
Age		14.4 (0.03)	14.4 (0.03)	14.4 (0.06)
Gender	Female	542 (47.6)	329 (47.4)	213 (48.2)
	Male	480 (52.4)	292 (52.6)	188 (51.8)
ECEC-start-age group	Below age 1	144 (10.3)	45 (7.3)	99 (22.9)
	Age 1–2	214 (13.4)	41 (6.1)	173 (43.9)
	Age 2–3	366 (41.1)	261 (44.3)	105 (27.9)
	Age 3 + and before schooling	205 (22.4)	185 (26.7)	20 (4.2)
	Only cared in Family before schooling	93 (12.8)	89 (15.6)	4 (1.1)
Psycho-social problems	SDQ Total Score	7.4 (0.20)	7.4 (0.24)	7.5 (0.27)
	Externalizing psycho-social problems	4.4 (0.14)	4.4 (0.17)	4.3 (0.15)
	Internalizing psycho-social problems	3.1 (0.11)	3.1 (0.13)	3.1 (0.18)
Social Economic Status	Low	93 (16.5)	47 (15.4)	46 (20.8)
	Middle	608 (57.3)	365 (57.5)	243 (56.3)
	High	314 (25.2)	203 (25.9)	111 (22.6)
	Missing	7 (1.0)	6 (1.2)	1 (0.4)
Migrant Status	Non-migrant	927 (83.3)	540 (81.6)	387 (90.1)
	Migrant	88 (16.0)	78 (17.8)	10 (8.5)
	Missing	7 (0.7)	3 (0.5)	4 (1.3)
Number of older siblings	0	206 (20.7)	132 (21.4)	74 (17.7)
	1	384 (37.3)	252 (38.4)	132 (32.7)
	2	127 (14.0)	86 (15.0)	41 (10.2)
	3 and more	45 (3.9)	26 (4.0)	19 (3.6)
	Missing	260 (24.1)	125 (21.3)	135 (35.8)
Family situation (With whom the child live together primarily?)	Natural parents in a joint household	910 (86.3)	572 (88.6)	338 (76.6)
	Mother or father with their own partner	25 (2.2)	9 (1.6)	16 (4.9)
	Mother or father without partner	73 (10.4)	36 (9.2)	37 (15.4)
	Others (grandparents etc.)	6 (0.3)	0 (0.0)	6 (1.7)
	Missing	8 (0.8)	4 (0.7)	4 (1.3)
Family cohesion	Min-<=20th Percentile	203 (21.0)	125 (20.4)	78 (23.1)
	20th - <=40th Percentile	205 (20.5)	134 (21.5)	71 (16.3)
	40th - <=60th Percentile	249 (23.6)	149 (24.0)	100 (21.8)
	60th - <=80th Percentile	189 (15.6)	109 (14.6)	80 (19.8)
	80th Percentile-Max	139 (14.1)	77 (13.7)	62 (15.7)
	Missing	37 (5.3)	27 (5.7)	10 (3.2)
Parents’ Divorce	Did	165 (16.7)	83 (15.0)	82 (23.0)
Birth weight	< 2500 g	57 (5.2)	28 (4.8)	29 (6.8)
	>=2500 g and < 4000 g	811 (78.8)	488 (78.0)	323 (81.8)
	>=4000 g	136 (12.9)	90 (13.5)	46 (10.6)
	Missing	18 (3.1)	15 (3.7)	3 (0.8)
Obesity/Overweight	Obesity	22 (2.0)	14 (2.2)	8 (1.4)
	Overweight but not obesity	59 (6.4)	37 (6.8)	22 (4.8)
	Neither obesity nor overweight	927 (89.5)	559 (88.7)	368 (92.6)
	Missing	14 (2.1)	11 (2.3)	3 (1.2)
Age of mother at childbirth	Till 24 years old	134 (17.1)	68 (15.4)	66 (23.8)
	25–29 years old	279 (25.2)	136 (23.5)	143 (32.1)
	30–34 years old	374 (35.5)	247 (36.8)	127 (30.1)
	35 + years old	212 (19.9)	158 (22.1)	54 (10.8)
	Missing	23 (2.3)	12 (2.1)	11 (3.2)
Employment status of mother	Full-time employed	194 (13.2)	53 (8.1)	141 (34.7)
	Part-time employed	382 (36.8)	253 (37.7)	129 (33.0)
	Unemployed	432 (47.7)	304 (51.6)	128 (31.4)
	Missing	14 (2.3)	11 (2.7)	3 (0.9)
Region (East/West Germany)	West Germany	621 (80.7)		
	East Germany (including Berlin)	401 (19.3)		
Schooling type^b^	Academic secondary school preparing for university	467 (38.0)	270 (36.3)	197 (45.3)
	Secondary school preparing for vocational training in trade	235 (24.0)	157 (25.4)	78 (18.4)
	Secondary general school preparing for vocational training in crafts	36 (5.9)	29 (7.0)	7 (1.5)
	Comprehensive school	133 (16.4)	88 (16.6)	45 (15.6)
	Secondary school preparing for vocational training in trade and crafts	98 (8.7)	39 (7.4)	59 (13.8)
	Others	35 (4.9)	23 (4.9)	12 (4.5)
	Missing	18 (2.1)	15 (2.4)	3 (0.8)

^a^The weighted means and percentages have been listed. Data of age, type of schooling, and parental divorce were assessed at wave 2; ECEC-start-age was derived using data from Baseline wave and wave 1; all the other data in this table were collected at baseline wave

^b^Schooling type in Germany:

Academic secondary school preparing for university (*Gymnasium*) is the highest form of secondary education and aims to prepare students for continued university education. The curriculum at a *Gymnasium* has an academic focus, with a minimum of two foreign languages, higher math, and science courses, with the goal to reach the university level

Secondary school preparing for vocational training in trade (*Realschule*) offers mid-level education. It is more challenging than the *Hauptschule*, but a step lower than the *Gymnasium*. The *Realschule* prepares students with practical and theoretical knowledge for their future professional life. Students usually have the option to choose a focus area, such as an additional foreign language or science subject

Secondary general school preparing for vocational training in crafts (*Hauptschule*) offers the lowest, least demanding learning level in the German education system. It is a choice for pupils who want to continue their education with an apprenticeship in crafts

Comprehensive school (*Gesamtschule*) combines all three tracks of school education (*Gymnasium*, *Realschule* and *Hauptschule*) in one comprehensive school, making it easier to switch tracks if necessary

Secondary school preparing for vocational training in trade and crafts (*Haupt-und Realschule*) combines *Realschule* and *Hauptschule* in one school

Most of the descriptive variables for baseline characters were similar for West and East Germany, with the exception of ECEC-start-age, mother’s employment status, SES, family structures and migrant status. More mothers with 3–4 year old children in East Germany were in full-time employment (34.7%) than in West Germany (8.1%), and fewer mothers in East Germany were unemployed (31.4%) than in West Germany (51.6%). The majority of children in West Germany started ECEC at the age of 2–3 years (44.3%), whereas the majority of children in East Germany started ECEC at the age of 1–2 years (43.9%). Only 7.3% of children in West Germany started ECEC younger than 1 year, while 22.9% of children in East Germany started younger than 1 year. In terms of SES, 15.4% of children in West Germany and 20.8% of children in East Germany came from low SES families, while 25.9% of children in West Germany and 22.6% of children in East Germany came from high SES families. In West Germany there were more family structure as natural parents in a joint household (88.6%) and more migrant (17.8%) than in East Germany (76.6% and 8.5% respectively).

Additional analysis showed that SES and family structure differed significantly among ECEC-start-age groups in West Germany whereas it didn’t in East Germany (Supplementary Material 7). Mother’s employment status when the child was 3–4 years old differed significantly among ECEC-start-age groups in both West and East Germany. Regarding children starting ECEC younger than 1 year in the West Germany, they tended to be more likely in a one-parent family with high SES and with a full-time employed mother.

**Fig. 2:**
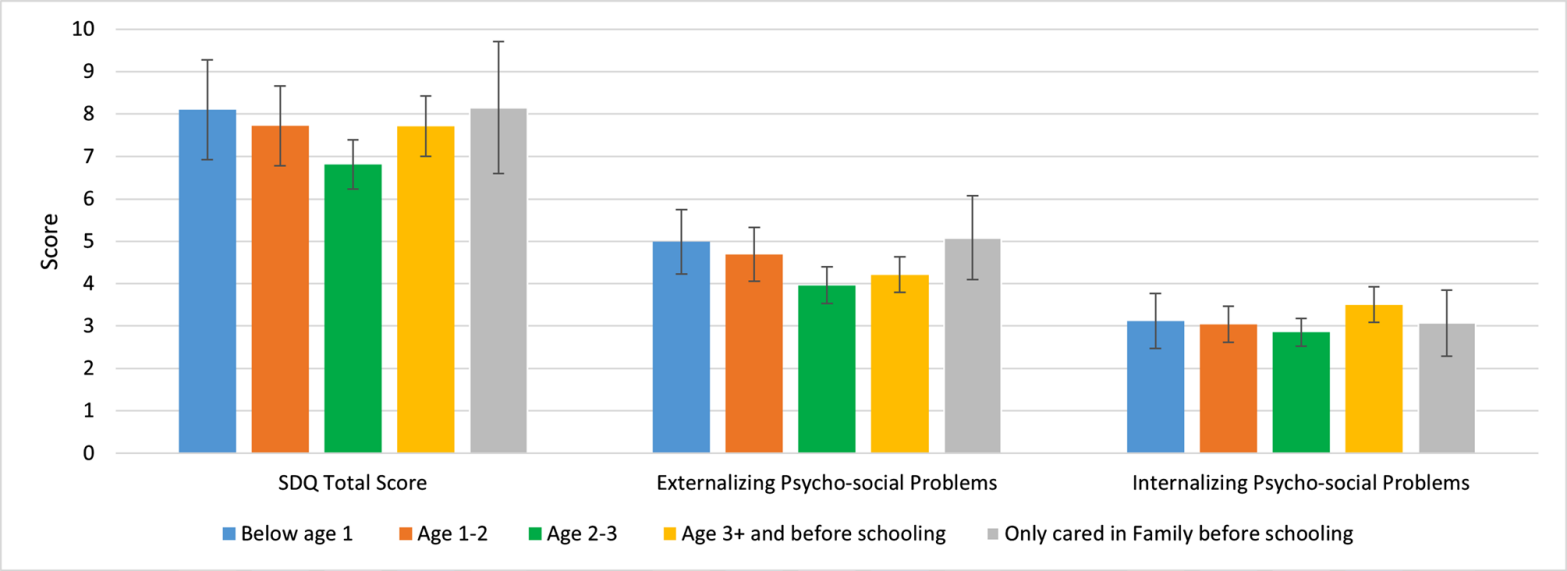
Mean values and 95% CI of psycho-social problems in adolescence by ECEC-start-age group (n=1022)

Figure 2 illustrates the mean values and 95% confidence intervals of psycho-social problems in adolescence by ECEC-start-age group in the whole sample. Compared to the other ECEC-start-age groups, those who started ECEC between the ages of 2 and 3 had the lowest SDQ scores, indicating the least psycho-social problems in adolescence (mean SDQ total score = 6.81; mean score of externalizing psycho-social problems = 3.96; mean score of internalizing psycho-social problems = 2.85). Those who started ECEC before 2 years of age had higher SDQ scores than children in the reference group (ECEC-start-age = “Age 2–3”), and the earlier the child started ECEC, the higher the SDQ scores were. Among them, those who started ECEC within the first year after birth had the highest total SDQ score (8.10) and the highest score for externalizing psycho-social problems (4.99). Those who started ECEC after the age of 3 (but before school entry) had the highest score for internalizing psycho-social problems (3.50).

**Fig. 3:**
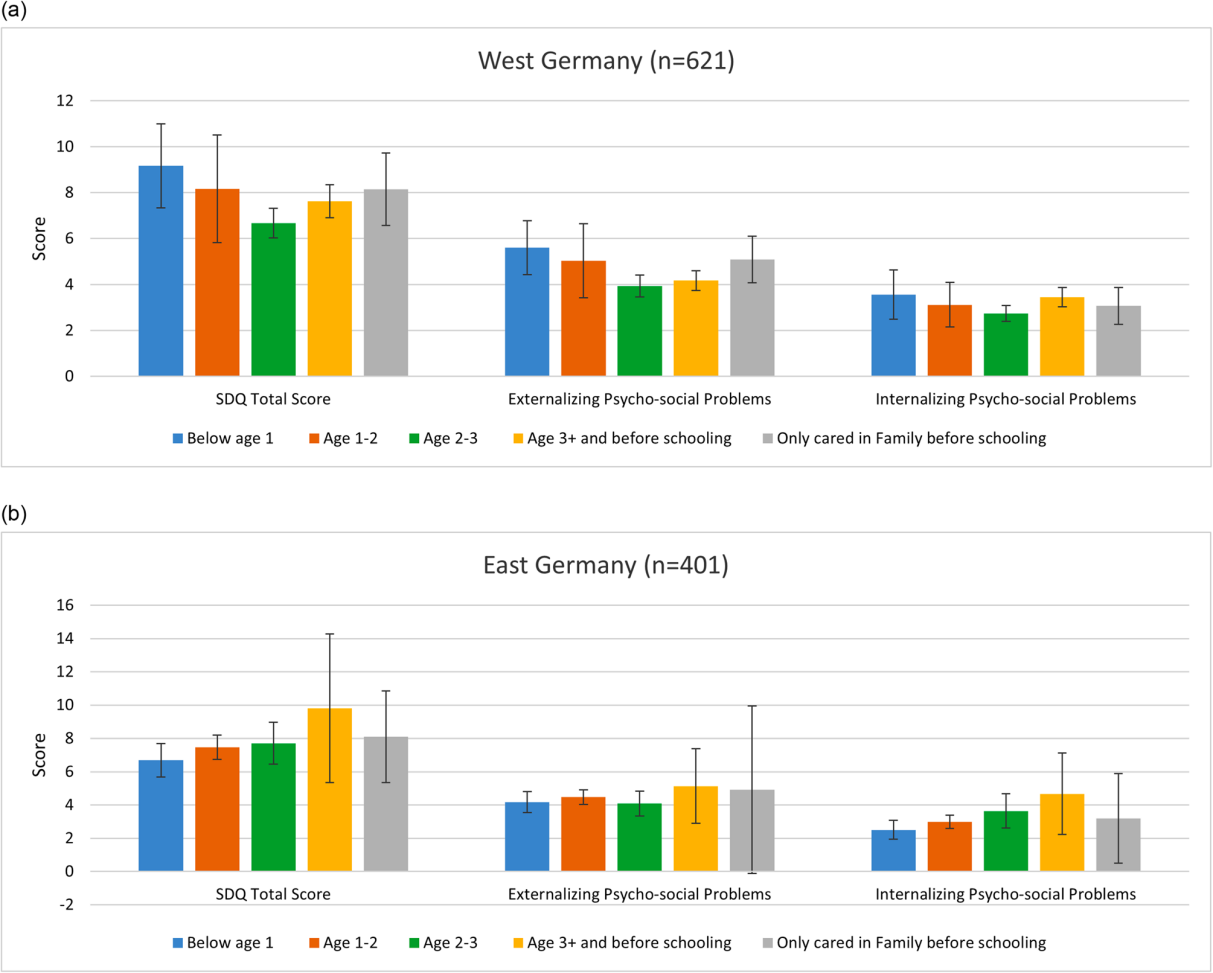
Mean values and 95% CI of psycho-social problems in adolescence by ECEC-start-age group and region. (**a**) Mean values and 95% CI of psycho-social problems in adolescence by ECEC-start-age group in West Germany. (**b**) Mean values and 95% CI of psycho-social problems in adolescence by ECEC-start-age group in East Germany

By region, the pattern in West Germany is similar to that for the whole Germany, whereas only externalizing psycho-social problems (except for the ECEC-start-age group < 1 year old) in East Germany is similar to that for the whole Germany (Fig. 3). In East Germany, the ECEC-start-age group < 1 year had lower scores for externalizing psycho-social problems than the group of “Age 1–2”; for internalizing psycho-social problems, the later children started the ECEC, the higher their scores in adolescence in general.

Our multivariate results (Table 2) show that, compared to the reference group (ECEC-start-age between the ages of 2–3), the total SDQ score was on average 1.8 units higher for those who started ECEC younger than 1 year (p = 0.002), 1.4 units higher for those who started ECEC between the ages of 1–2 (p = 0.036), and 1.0 unit higher for those who started ECEC older than 3 years old but before starting school (p = 0.020). Those starting ECEC younger than 2 years had statistically significant higher scores for externalizing psycho-social problems (beta = 1.3, p = 0.001 for “Below age 1”; beta = 1.2, p = 0.005 for “Age 1–2”). Those who started ECEC older than 3 years old (but before starting school) had statistically significantly higher scores for internalizing psycho-social problems (beta = 0.7, p = 0.017).

**Table 2:** Linear regression of the association between ECEC-start-age and psycho-social problems in adolescence after controlling for confounders (n = 1022)

	ECEC-start-age groups		SDQ Total Score			Externalizing Psycho-social Problems			Internalizing Psycho-social Problems	
		n	Beta (95%CI)	P		Beta (95%CI)	P		Beta (95%CI)	P
Germany (n = 1022)	Below age 1	144	**1.8 (0.6,2.9)**	0.002*		**1.3 (0.6,2.0)**	0.001*		0.5 (-0.3,1.2)	0.245
	Age 1–2	214	**1.4 (0.1,2.8)**	0.036*		**1.2 (0.4,2.1)**	0.005*		0.2 (-0.5,0.9)	0.586
	Age 2–3 (Reference group)	366								
	Age 3 + and before schooling	205	**1.0 (0.2,1.9)**	0.020*		0.4 (-0.1,0.9)	0.149		**0.7 (0.1,1.2)**	0.017*
	Only cared in Family before schooling	93	0.8 (-0.6,2.1)	0.255		0.7 (-0.1,1.6)	0.099		0.0 (-0.7,0.8)	0.930

Covariates: age in adolescence, gender, schooling type, parents’ divorce by wave 2, and other baseline characters including region (East/West Germany), social economic status, migrant status, number of older siblings, family situation, family cohesion, birth weight, obesity/overweight, age of mother at childbirth, and employment status of mother

* p < 0.05

In West Germany, overall, results were comparable to the findings for whole Germany (Table 3). The only exception was the ECEC-start-age group of “Age 1–2”. While this ECEC-start-age group showed statistically significant increased SDQ score (beta = 1.4, p = 0.036) in the whole Germany, this group showed a tendency of increased SDQ score (beta = 2.3, but statistically insignificant with p = 0.055) in West Germany. However, for the externalizing psycho-social problems, children starting ECEC younger than 2 years had statistically significant higher scores in the whole Germany and in West Germany as well.

**Table 3:** Stratified Linear regression of the association between ECEC-start-age and psycho-social problems in adolescence after controlling for confounders by region (West/East Germany) (n = 1022)

	ECEC-start-age groups		SDQ Total Score			Externalizing Psycho-social Problems			Internalizing Psycho-social Problems	
		n	Beta (95%CI)	P		Beta (95%CI)	P		Beta (95%CI)	P
West Germany (n = 621)	Below age 1	45	**2.9 (1.3,4.6)**	< 0.001*		**1.9 (0.9,3.0)**	< 0.001*		1.0 (-0.2,2.1)	0.088
	Age 1–2	41	2.3 (-0.1,4.6)	0.055		**1.7 (0.2,3.3)**	0.029*		0.6 (-0.5,1.6)	0.300
	Age 2–3 (Reference group)	261								
	Age 3 + and before schooling	185	**1.2 (0.4,2.1)**	0.006*		0.4 (-0.1,1.0)	0.094		**0.8 (0.2,1.4)**	0.005*
	Only cared in Family before schooling	89	0.9 (-0.5,2.3)	0.214		0.8 (-0.2,1.7)	0.108		0.1 (-0.6,0.9)	0.731
East Germany (n = 401)	Below age 1	99	0.3 (-1.3,1.9)	0.714		0.3 (-0.5,1.1)	0.514		0.0 (-1.1,1.1)	0.971
	Age 1–2	173	0.4 (-1.0,1.8)	0.589		0.6 (-0.03,1.3)	0.062		-0.3 (-1.3,0.7)	0.609
	Age 2–3 (Reference group)	105								
	Age 3 + and before schooling	20	2.1 (-2.4,6.6)	0.363		0.8 (-1.2,2.8)	0.406		1.2 (-1.6,4.1)	0.394
	Only cared in Family before schooling	4	-1.4 (-5.1,2.4)	0.462		-0.6 (-2.0,0.7)	0.341		-0.7 (-4.0,2.5)	0.648

Covariates: age in adolescence, gender, schooling type, parents’ divorce by wave 2, and other baseline characters including social economic status, migrant status, number of older siblings, family situation, family cohesion, birth weight, obesity/overweight, age of mother at childbirth, and employment status of mother

* p < 0.05

In East Germany, in contrast, the analysis revealed no statistically significant differences depending on ECEC starting age (Table 3). Those who started ECEC at the age of 1–2 years tended to have higher scores for externalizing psycho-social problems in East Germany, but these association was statistically insignificant at the conventional significance level p = 0.05 (beta = 0.6, p = 0.062). The beta of “Age 3 + and before schooling” for SDQ total score and for internalizing psycho-social problems were relatively high (2.1 and 1.2), and the sample size for this group of ECEC-start-age was relatively small (n = 20). The p-values for these two betas were statistically insignificant (p = 0.363 and p = 0.394).

The linear regression with an interaction factor of ECEC-start-age by region shows a statistically significant interaction of ECEC-start-age younger than 1 year by region on SDQ total score (p = 0.001), on externalizing psycho-social problems (p = 0.016) and on the internalizing psycho-social problems (p = 0.018) (see Supplementary Material 4). There was an unfavorable effect of ECEC younger than 1 year in West Germany but not East Germany. No such statistically significant interaction was observed for other ECEC-start-ages by region at the conventional significance level p = 0.05 (see Supplementary Material 4).

Sensitivity analysis confirmed our results which remained robust after dropping outliers (n for outliers = 76) (Supplementary Material 5). The plots to check the assumptions of linear regression are also provided in Supplementary Material 6.

## Discussion

Starting ECEC later than 3 years of age is associated with higher parent-reported scores of internalizing psycho-social problems in adolescence in both West Germany (with statistically significant results) and East Germany (with a relatively larger effect size but statistically insignificant results). For externalizing psycho-social problems, starting ECEC between the ages of 2 and 3 is associated with the lowest score; in West Germany, those who start before the age of 2 have more externalizing psycho-social problems, and this disadvantage increases with an even younger starting age. However, in East Germany only those who start ECEC between 1 and 2 years old tend to have higher scores of externalizing psycho-social problems. This association does not hold in East Germany for the children starting ECEC younger than 1 year old. The regional difference in the results for children under 1 year of age may suggest a selection effect in West Germany where only fewer parents use this option.

A major limitation of previous observational studies investigating the effect of ECEC is the possibility of a selection bias due to unmeasured or unknown potential confounders which cannot be fully addressed by conventional statistical approaches [27, 28]. Our study uses a natural experiment with similar family policies and a number of other similarities such as the education system and language within Germany, but with a different cultural context regarding the role of women and consequently different use and availability of ECEC in West and East Germany. Thus, our study is able to reveal the selection effect which most observational studies with conventional statistical approach are not able to do. A previous study from Norway has also successfully applied the natural experiment study design by using birth month as an additional factor of interest when exploring the association between ECEC and aggression [29].

### Starting ECEC younger than 1 year old: inconsistent association in West and East Germany

Children starting ECEC younger than 1 year old had a higher possibility of psycho-social problems in West Germany, but not in East Germany. Selection processes may be at work that leads to this disadvantage for children starting ECEC younger than 1 in terms of psycho-social developments in West Germany. Our data showed that the children starting ECEC younger than 1 in the West Germany were more likely from a one-parent high-SES family with a full-time employed mother.

The regional culture difference regarding the role of women and consequently regarding the attitude to the ECEC is worth discussing. In West Germany, the mothers who bring the children to the ECEC earlier may get stress or even blame from relatives or neighbours; in East Germany, the children who get parental care at home may regard themselves strange if the majority of their peer friends go to an ECEC. This culture difference may be one of the sources of the possible selection effects for the children starting ECEC younger than 1 year in West Germany. Concretely, in West Germany, families with “problems” (e.g., job overload, partnership problems, lack of social support by grandparents or friends, economic problems) may be more or less forced to bring children to non-parental ECEC in the first life year; and these “problems” might be the actual reason(s) for more psycho-social difficulties in children’s development. In East Germany, in contrast, it is more accepted or normal to bring children to non-parental ECEC at this young age, even in “non-problematic” families.

Further research is needed to identify the sources of this possible selection process.

### Starting ECEC between 1 and 2 years old: increased probability of externalizing psycho-social problems

It is interesting to note that in both West and East Germany there is a tendency towards increased externalizing psycho-social problems for those who start ECEC between the ages of 1 and 2. Teachers and parents therefore need to pay more attention to preventing children in this group of ECEC-start-age from developing externalizing psycho-social problems. Previous studies have reported that prolonged exposure to ECEC before age 2 was associated with a higher risk of behavioral problems and long-term insecure attachment, but short exposure to ECEC did not have such negative effects [10, 30]. Therefore, participation in ECEC on a part-time basis may be a compromise but better option for parents who wish to work at an earlier stage in the child’s life. The quality of ECEC and the qualifications of nursery teachers need to be carefully and regularly monitored, in order to maintain a good teacher-child ratio, and to provide stable, continuous and sensitive care for children, which may help to prevent early insecure attachment and, consequently, externalizing psycho-social problems [10, 16].

### Starting ECEC at 3 + years old: increased probability of internalizing psycho-social problems

We observed an increased probability of internalizing psycho-social problems in the group of children starting ECEC over the age of 3 years in both West Germany (with statistically significant results) and East Germany (with a relatively larger effect size but statistically insignificant results). The small sample size (n = 20) in East Germany may explain the lack of statistical significance. This observation underlines the importance of participating in ECEC at the age of 3 years at the latest. Interestingly, Fig. 3 shows a “dosage effect” of ECEC-start-age on the increased probability of internalizing psycho-social problems from ECEC-start-age < 1 to 3 + in East Germany. The later the children started ECEC, the higher the probability that the children will have internalizing psycho-social problems in adolescence.

The importance of playing with peers in the pre-school age group, from three years of age onwards, may explain this association. At these ages, playing with others is the central activity of children [31, 32]. Playing and interacting with their peers at these ages is a good way to explore and develop their social, emotional, language and cognitive skills. Social and cooperative play helps children develop a sense of initiative and confidence in their ability to make decisions, solve problems and influence others [31, 32]. Participating in ECEC provides good opportunities to play and interact with peers. In addition, having no close friends may increase the risk of internalizing psycho-social problems [17]. Therefore, if a child doesn’t start ECEC at the age of 3, more attention should be paid to preventing internalizing psycho-social symptoms, for example by providing more opportunities to play with peers.

## Strengths and limitations

Our study has several strengths. Our study has used a nationally representative and adequately large sample of children and their parents in Germany. We have considerable variations of ECEC-start-age in the data, and explored the similarity and difference of the association between West and East Germany in a natural experiment. Thus, we provide more socio-political variations and contribute to the external validity in this research field. Furthermore, by using the natural experiment, we have found the potential selection effect to interpret the negative association between ECEC and psycho-social development for the children starting ECEC younger than 1 in West Germany. Moreover, we have examined the pattern of ECEC-start-age rather than just using a cut-off point for ECEC-start-age. By doing so, we are able to show that the association is not linear, and to reveal both the beneficial and unfavourable association between ECEC-start-age and psycho-social development in adolescence. Furthermore, most previous studies used discrete categories to analyse psycho-social problems [8]. Our study analyses psycho-social problems using a continuous score, which may provide a more valid and reliable assessment [8]. However, further research is still needed to use discrete categories to analyse these associations, to support the clinical relevance of these associations. Last but not least, we have found the association between ECEC-start-age and psycho-social problems in adolescence. There is at least 10 years between the exposure of interest and the outcome of interest. We are helping to provide evidence to fill the gap in the literature.

Our study also has some limitations. Firstly, the quality and intensity of ECEC exposure influences the association [33]. However, neither quality nor intensity data on ECEC exposure are available in KiGGS. Further research is needed to include these different aspects in the analysis. Secondly, some factors which may influence the association have not been considered, such as later school experience and the relationship between parents and child. However, we have controlled for some of the potential confounders, such as type of schooling and family cohesion, in our analysis to reduce this kind of bias. The natural experimental design of the study also helps to reveal potential unmeasured or unknown biases. However, not all of these biases can be fully controlled for. These factors should be investigated in more detail in further studies. Thirdly, there may be an attrition bias. However, we have used longitudinal weights to minimize this bias. Furthermore, we have used parent-reported SDQ scores in our analysis, which may differ from self or teacher-reported scores. However, the German parent SDQ has been shown to have reliable and useful psychometric properties [34]. Further analysis using self or teacher-reported measures in the future may provide more insight into this research question. Moreover, as a general limitation of observational studies, our results are associations rather than causalities. Therefore, the results should be interpreted with caution. However, our findings remain valuable because our findings suggest that more attentions from teachers and parents should be given to some particular psycho-social problems for some particular ECEC-start-age groups. Finally, the sample size for the ECEC-start-age group of ‘only cared for in family before schooling’ is relatively small (n = 93), which may limit the statistical power for this group.

## Conclusion

The regional similarities between West and East Germany with regard to the increased probability of internalizing psycho-social problems among those starting ECEC at the age of 3 or older indicate the importance of providing access to ECEC for these children, while the regional difference with regard to the probability of psycho-social problems among those starting ECEC at the age of 1 year or younger suggests a possible selection effect for the observed association in West Germany, where fewer parents use this option. However, in both West and East Germany, children starting ECEC between the ages of 1 and 2 tend to have a higher possibility of externalizing psycho-social problems. In conclusion, for those who start ECEC older than 3 years old, more attention may be needed to prevent potential internalizing psycho-social problems, for example by providing more opportunities for social interaction through peer play. For those who start ECEC younger than 2 years old, more attention by parents and nursery teachers is needed to prevent potential externalizing psycho-social problems. A stable and continuous relationship with nursery teachers as well as a not too long hours of daily care could be helpful. For those who start ECEC younger than 1 year old, more research is needed to better understand why children in West Germany are particularly disadvantaged and those in East Germany are not.

### Electronic supplementary material

Below is the link to the electronic supplementary material.

**Additional file 1**: **1**. Operationalization of ECEC-start-age. **2**. Operationalization of potential confounders. **3**. Potential confounders: association with exposure and outcome of interest. **4**. Linear Regression with Interaction Factor Region*ECEC-start-age. **5**. Sensitivity analysis for the outliers. **6**. Diagnostics for linear regression. **7**. Distribution of SES and other Covariates by ECEC-start-age in West and East Germany. **8**. Table to support Figures 2 and 3 in the main paper

## Data Availability

The authors confirm that some access restrictions apply to the data underlying the findings. The data set cannot be made publicly available because informed consent from study participants did not cover public deposition of data. However, the minimal data set underlying the findings is archived in the ‘Health Monitoring’ Research Data Centre at the Robert Koch Institute (RKI) and can be accessed by researchers on reasonable request. On-site access to the data set is possible at the Secure Data Center of the RKI’s ‘Health Monitoring’ Research Data Centre. Requests should be submitted to the ‘Health Monitoring’ Research Data Centre, Robert Koch Institute, Berlin, Germany (e-mail: fdz@rki.de).
