# Supplementary material for: Association between the starting age of non-parental Early Childhood Education and Care (ECEC), and psycho-social problems in adolescence in West and East Germany – a natural experiment using data from the German Health Interview and Examination Survey for Children and Adolescents (KiGGS)
Source: BMC Psychol. 2023 Nov 20;11:403. doi: 10.1186/s40359-023-01447-1 (PMC10658975; doi:10.1186/s40359-023-01447-1)
Supplement: Supplementary file 1 — Additional file 1: 1. Operationalization of ECEC-start-age. 2. Operationalization of potential confounders. 3. Potential confounders: association with exposure and outcome of interest. 4. Linear Regression with Interaction Factor Region*ECEC-start-age. 5. Sensitivity analysis for the outliers. 6. Diagnostics for linear regression. 7. Distribution of SES and other Covariates by ECEC-start-age in West and East Germany. 8. Table to support Figures 2 and 3 in the main paper [file 40359_2023_1447_MOESM1_ESM.docx]

Contents

[1. Operationalization of ECEC-start-age 2](#_Toc150633128)

[2. Operationalization of potential confounders 2](#_Toc150633129)

[3. Potential confounders: association with exposure and outcome of interest 4](#_Toc150633130)

[4. Linear Regression with Interaction Factor Region*ECEC-start-age 6](#_Toc150633131)

[5. Sensitivity analysis for the outliers 7](#_Toc150633132)

[6. Diagnostics for linear regression 8](#_Toc150633133)

[7. Distribution of SES and other Covariates by ECEC-start-age in West and East Germany 10](#_Toc150633134)

[8. Table to support Figures 2 and 3 in the main paper 13](#_Toc150633135)

[Reference 14](#_Toc150633136)

# 1. Operationalization of ECEC-start-age

ECEC-start-age was derived through the following rules:

a. If the age for beginning ECEC at wave 2003-2006 was available, then ECEC-start-age = age for beginning ECEC at wave 2003-2006.

b. Else if the age for beginning ECEC was available at wave 2009-2012, then ECEC-start-age = age for beginning ECEC from wave 2009-2012.

c. Else if the children were only cared for in the family in both waves (baseline and wave 1), then ECEC-start-age = “Care only in family before schooling”.

d. Else if the answers to the question “Was or is your child only be cared for in the family before schooling” at the two waves were inconsistent, then children were excluded from the analysis to ensure high validity of the exposure variable.

# 2. Operationalization of potential confounders

***SES***

The scores of education, occupation and income per household were summed up at first, and then the sum was classified by quintiles into low SES (first quintile), medium SES (second to fourth quintile) and high SES (fifth quintile) (see (1) for details).

***Migration status***

The migration status was categorized as non-migrant and migrant. A child was defined as migrant if she/he immigrated from another country and at least one of her/his parents was not born in Germany, or if both of her/his parents had a foreign citizenship or immigrated from another country (2).

***Number of older siblings (Order of birth)***

Number of older siblings presented how many brothers or sisters were older than the child who participated in KiGGS. That number plus 1 reflected the order of birth for the child participating in KiGGS among siblings in the family.

***Family situation and family cohesion***

The family situation was categorized as: natural parents in a joint household, mother or father with their own partner, mother or father without partner and others. Additionally, a variable is included that indicates whether the parents experienced divorce by the survey time of wave 2.

As an indicator for the extent of emotional support from the family members, the subscale “family cohesion” was used from the family climate scale (3). The subscale “family cohesion” comprises four items by using 4-stage options from “definitely no” to “definitely yes”. In the analysis, the sum value over the four items is categorized by using 20 percentile, so that the subjects with missing values can remain in the analysis.

***Birth weight***

Birth weight was categorized as <2500g, >=2500g and <4000g, and >=4000g (4).

***Obesity/overweight***

Obesity/Overweight of the child was categorized as obese, overweight without obesity, neither obese nor overweight. Normal weight (≤90th percentile, P90), overweight (>P90 to ≤P97) and obesity (>P97) were defined using the national German percentiles (5).

***Age of mother at childbirth***

Age of mother at childbirth was categorized as the group till 24 years of age, 25-29 years old, 30-34 years old and 35 years old and older (35+).

***Employment status of mother***

Employment status of mother was categorized as full-time employed, part-time employed (including mini-job) and unemployed (including housewife, or due to study, retirement or parental leave etc.).

***Region (east/west*** ***Germany)***

To account for the varying cultural context and supply of ECEC in east and west Germany, a regional variable differentiating between east (including Berlin) and west Germany was included.

***Schooling type***

Schooling type was categorized as “ Academic secondary school preparing for university“, “Secondary school preparing for vocational training in trade“, “Secondary general school preparing for vocational training in crafts“, “Comprehensive school“, “Secondary school preparing for vocational training in trade and crafts “ and others at wave 2. Academic Secondary School preparing for university (*Gymnasium*) is the highest form of secondary education and aims to prepare students for continued university education. The curriculum at a *Gymnasium* has an academic focus, with a minimum of two foreign languages, higher math, and science courses, with the goal to reach the university level. Secondary School preparing for vocational training in trade (*Realschule*) offers mid-level education. It is more challenging than the *Hauptschule*, but a step lower than the *Gymnasium*. The *Realschule* prepares students with practical and theoretical knowledge for their future professional life. Students usually have the option to choose a focus area, such as an additional foreign language or science subject. Secondary General School preparing for vocational training in crafts (*Hauptschule*) offers the lowest, least demanding learning level in the German education system. It is a choice for pupils who want to continue their education with an apprenticeship in craft or industrial trades. Comprehensive School (*Gesamtschule*) combines all three tracks of school education (i.e. *Gymnasium, Realschule* and *Hauptschule*) in one comprehensive school, making it easier to switch tracks if necessary. Secondary School preparing for vocational training in trade and crafts (*Haupt-u. Realschule*) combines Realschule and Hauptschule in one school.

# 3. Potential confounders: association with exposure and outcome of interest

**Table S1. Previous findings regarding potential confounders and their association with ECEC-participation and psycho-social problems**

| **Potential confounder** | **Associated with ECEC-participation** | | **Risk or predictor factors for psycho-social problems** | |
| --- | --- | --- | --- | --- |
|  | **Previous findings** | **Reference** | **Previous findings** | **Reference** |
| SES | Household income, education and employment biography (of the mother) are associated with ECEC-participation | (6) | Social-demographic factors (low SES) is such a factor | (7, 8) |
| Migration Status | Migrant background is associated with ECEC-participation | (9) | Migrant background is such a factor | (2) |
| Number of Older Siblings (Order of birth) | Subsequent siblings within one family have a higher priority for an ECEC place compared to the first or single child in Germany when demand of ECEC places exceeds supply. | (10) | Birth order is such a factor | (11) |
| Family Situation | Household structure (single-parent or two-parent families etc.) is associated with ECEC-participation | (12) | Single-parent family is such a factor | (8) |
| Family Cohesion | Family cohesion has a positive association with school belonging, mediated by security and achievement goals. Moreover, family cohesion is associated with household structure. | (13) | Lack of family cohesion is such a factor | (8) |
| Parents’ Divorce | Household structure (single-parent or two-parent families etc.) is associated with ECEC-participation | (12) | Parents’ divorce results in psycho-social and mental problems among their children | (14) |
| Birth Weight | A factor influencing ECEC-participation may be the birth weight, because parents of children with low birth weight return to work less or later or work less than parents with a normal birthweight child | (15) | Low birth weight is such a factor | (16) |
| Obesity/Overweight | It could be hypothesized that children in ECEC profit from more physical activities and healthier food and hence have a lower likelihood of being overweight/obese. An evidence is that school closure during COVID-19 pandemic promotes childhood obesity | (17) | Obesity or overweight is such a factor | (18) |
| Age of mother at childbirth | Advanced maternal age may be associated with low birth weight, which may influence ECEC-participation | (19, 15) | Advanced maternal age may be associated with low birth weight, which may be a risk/predictor factor for psycho-social problems | (19, 16) |
| Employment status of mother | Employment biography (of the mother) is associated with ECEC-participation | (6) | Parental unemployment is such a factor | (8) |
| Region (East/West Germany) | Belonging to east (“new” federal states) or west Germany (“old” federal states) is associated with ECEC-participation. Concretely, from 2002 to 2019 the use of ECEC for children under 3 years old increased from 37% to 52% in east Germany, and from 3% to 30% in west Germany | (20, 21) | Some social changes (e.g. economic stability, unemployment rate) may influence psycho-social problems. In Germany, these social changes differ in east and west Germany. | (7, 22) |
| Schooling Type | Schooling type at the school age is associated with ECEC-participation | (6) | Schooling type is such a factor | (8, 23) |

# 4. Linear Regression with Interaction Factor Region*ECEC-start-age

**Table S2. With interaction factor Region*ECEC-start-age: linear regression of the association between ECEC-start-age and psycho-social problems in adolescence after controlling for confounders (n=1022)**

|  | ECEC-start-age groups | SDQ Total Score | |  | Externalizing Psycho-social Problems | |  | Internalizing Psycho-social Problems | |
| --- | --- | --- | --- | --- | --- | --- | --- | --- | --- |
|  |  | Beta (95%CI) | P |  | Beta (95%CI) | P |  | Beta (95%CI) | P |
| Germany  (n=1022) | Below age 1 | **2.9 (1.4, 4.4)** | **<0.001*** |  | **1.8 (0.8, 2.8)** | **<0.001*** |  | 1.1 (-0.02, 2.1) | 0.054 |
|  | Age 1-2 | **2.3 (0.004, 4.6)** | **0.0496*** |  | **1.7 (0.2, 3.2)** | **0.030*** |  | 0.6 (-0.4, 1.7) | 0.238 |
|  | Age 2-3 (Reference group) |  |  |  |  |  |  |  |  |
|  | Age 3+ and before schooling | **1.1 (0.2, 2.0)** | **0.013*** |  | 0.4 (-0.1, 0.9) | 0.124 |  | **0.7 (0.2, 1.3)** | **0.011*** |
|  | Only cared in Family before schooling | 1.0 (-0.4, 2,4) | 0.178 |  | 0.8 (-0.1, 1.7) | 0.080 |  | 0.1 (-0.6, 0.9) | 0.736 |
|  |  |  |  |  |  |  |  |  |  |
|  | East Germany | 0.9 (-0.5, 2.3) | 0.204 |  | 0.1 (-0.8, 1.0) | 0.799 |  | 0.8 (-0.2, 1.8) | 0.128 |
|  | West Germany (Reference group) |  |  |  |  |  |  |  |  |
|  |  |  |  |  |  |  |  |  |  |
|  | Below age 1 * East | **-3.6 (-5.7, -1.4)** | **0.001*** |  | **-1.7 (-3.0, -0.3)** | **0.016*** |  | **-1.9 (-3.4, -0.3)** | **0.018*** |
|  | Age 1-2 * East | -2.5 (-5.2, 0.2) | 0.064 |  | -1.2 (-3.0, 0.5) | 0.167 |  | -1.3 (-2.7, 0.1) | 0.078 |
|  | Age 2-3 (Reference group) * East |  |  |  |  |  |  |  |  |
|  | Age 3+ and before schooling * East | 1.2 (-3.2, 5.6) | 0.595 |  | 0.7 (-1.4, 2.8) | 0.518 |  | 0.5 (-2.3, 3.3) | 0.724 |
|  | Only cared in Family before schooling* East | -3.1 (-6.8, 0.7) | 0.108 |  | -1.2 (-3.6, 1.2) | 0.312 |  | -1.8 (-4.9, 1.2) | 0.227 |

‘* p<0.05, Age 2-3 and West Germany are the reference group. Covariates: age in adolescence, gender, schooling type, parents’ divorce by wave 2, and other baseline characters including region (East/West Germany), social economic status, migrant status, number of older siblings, family situation, family cohesion, birth weight, obesity/overweight, age of mother at childbirth, employment status of mother. Interaction factor: region*ECEC-start-age

# 5. Sensitivity analysis for the outliers

**Table S3.1. Without outliers: linear regression of the association between ECEC-start-age and psycho-social problems in adolescence after controlling for confounders (n=946)^1^**

|  | ECEC-start-age groups |  | SDQ Total Score | |  | Externalizing Psycho-social Problems | |  | Internalizing Psycho-social Problems | |
| --- | --- | --- | --- | --- | --- | --- | --- | --- | --- | --- |
|  |  | n | Beta (95%CI) | P |  | Beta (95%CI) | P |  | Beta (95%CI) | P |
| Germany  (n=946) | Below age 1 | 130 | **1.2 (0.3,2.2)** | **0.012*** |  | **1.0 (0.3,1.7)** | **0.005*** |  | 0.2 (-0.5,0.9) | 0.570 |
|  | Age 1-2 | 200 | **1.0 (0.1,1.8)** | **0.036*** |  | **0.7 (0.2,1.2)** | **0.007*** |  | 0.2 (-0.3,0.8) | 0.398 |
|  | Age 2-3 (Reference group) | 340 |  |  |  |  |  |  |  |  |
|  | Age 3+ and before schooling | 189 | **0.8 (0.1,1.5)** | **0.033*** |  | 0.2 (-0.2,0.6) | 0.371 |  | **0.6 (0.2,1.0)** | **0.009*** |
|  | Only cared in Family before schooling | 87 | 0.7 (-0.3,1.7) | 0.176 |  | 0.6 (-0.02,1.3) | 0.059 |  | 0.0 (-0.6,0.7) | 0.913 |

^1.^ ‘* p<0.05, Outlier is defined as “values more than 2 standardized residuals away from zero in the linear regression”. Covariates: age in adolescence, gender, schooling type, parents’ divorce by wave 2, and other baseline characters including region (East/West Germany), social economic status, migrant status, number of older siblings, family situation, family cohesion, birth weight, obesity/overweight, age of mother at childbirth, and employment status of mother.

|  | ECEC-start-age groups |  | SDQ Total Score | |  | Externalizing Psycho-social Problems | |  | Internalizing Psycho-social Problems | |
| --- | --- | --- | --- | --- | --- | --- | --- | --- | --- | --- |
|  |  | n | Beta (95%CI) | P |  | Beta (95%CI) | P |  | Beta (95%CI) | P |
| West Germany  (n=571) | Below age 1 | 38 | **2.0 (0.7,3.4)** | **0.003*** |  | **1.7 (0.7,2.7)** | **0.001*** |  | 0.3 (-0.7,1.3) | 0.504 |
|  | Age 1-2 | 35 | 0.5 (-0.9,2.0) | 0.466 |  | 0.5 (-0.3,1.4) | 0.190 |  | 0.0 (-0.9,0.9) | 0.986 |
|  | Age 2-3 (Reference group) | 245 |  |  |  |  |  |  |  |  |
|  | Age 3+ and before schooling | 170 | **0.8 (0.03,1.6)** | **0.043*** |  | 0.2 (-0.3,0.7) | 0.371 |  | **0.6 (0.1,1.1)** | **0.013*** |
|  | Only cared in Family before schooling | 83 | 0.6 (-0.4,1.7) | 0.239 |  | 0.6 (-0.1,1.3) | 0.083 |  | 0.0 (-0.6,0.7) | 0.955 |
| East Germany  (n=375) | Below age 1 | 92 | 0.4 (-0.9,1.7) | 0.572 |  | 0.1 (-0.7,1.0) | 0.740 |  | 0.2 (-0.6,1.1) | 0.587 |
|  | Age 1-2 | 165 | 1.0 (-0.3,2.2) | 0.131 |  | **0.7 (0.05,1.4)** | **0.036*** |  | 0.2 (-0.6,1.1) | 0.549 |
|  | Age 2-3 (Reference group) | 95 |  |  |  |  |  |  |  |  |
|  | Age 3+ and before schooling | 19 | 2.1 (-0.7,4.9) | 0.140 |  | 0.7 (-1.0,2.4) | 0.434 |  | 1.5 (-0.1,3.0) | 0.059 |
|  | Only cared in Family before schooling | 4 | 0.7 (-1.3,2.8) | 0.480 |  | -0.1 (-1.2,1.0) | 0.819 |  | 0.8 (-1.2,2.8) | 0.399 |

**Table S3.2. Without outliers: Stratified Linear regression of the association between ECEC-start-age and psycho-social problems in adolescence after controlling for confounders by Region (West/East Germany) (n=946)^1^**

^1.^ ‘* p<0.05, Outlier is defined as “values more than 2 standardized residuals away from zero in the linear regression”. Covariates: age in adolescence, gender, schooling type, parents’ divorce by wave 2, and other baseline characters including social economic status, migrant status, number of older siblings, family situation, family cohesion, birth weight, obesity/overweight, age of mother at childbirth, and employment status of mother.

# 6. Diagnostics for linear regression


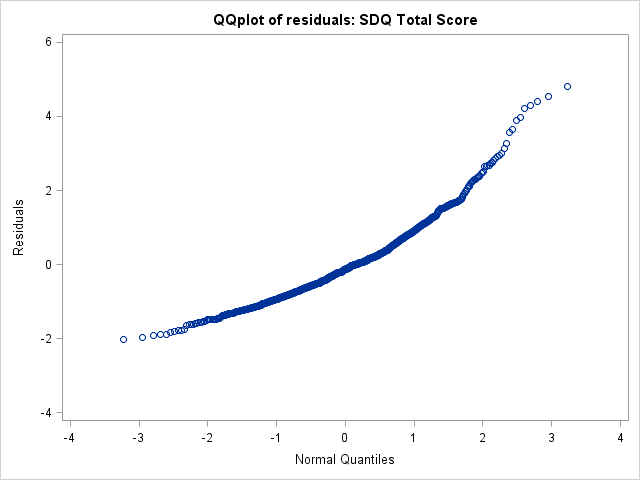

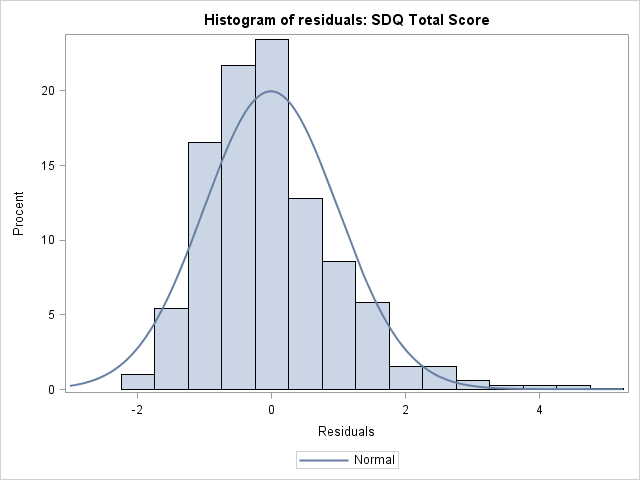
**Graphic S1.1. SDQ Total Score in Germany**


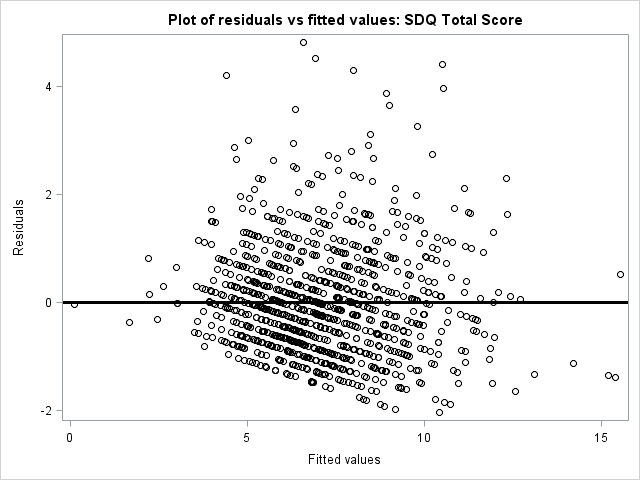


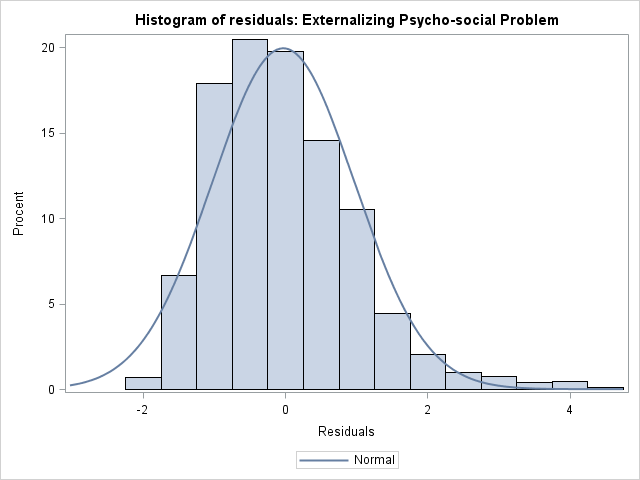
**
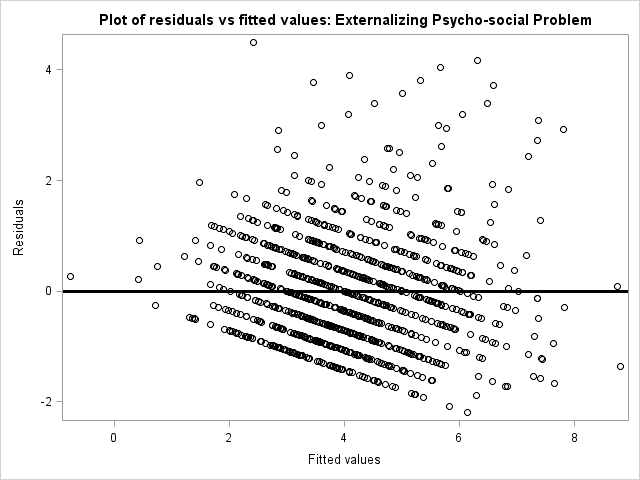
**
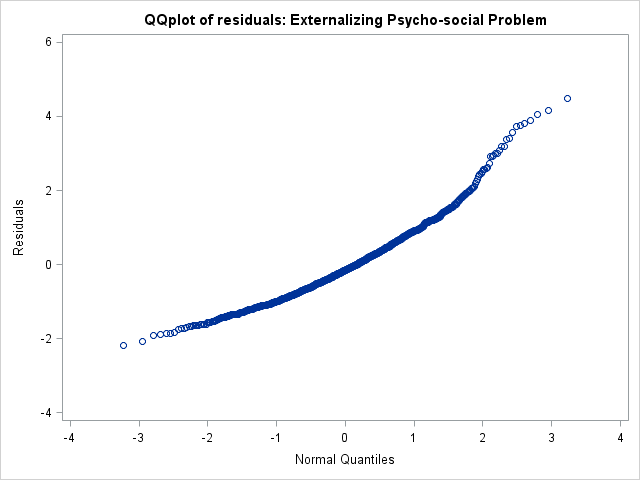
**Graphic S1.2. Externalizing Psycho-social Problems in Germany**

**Graphic S1.3. Internalizing Psycho-social Problems in Germany**


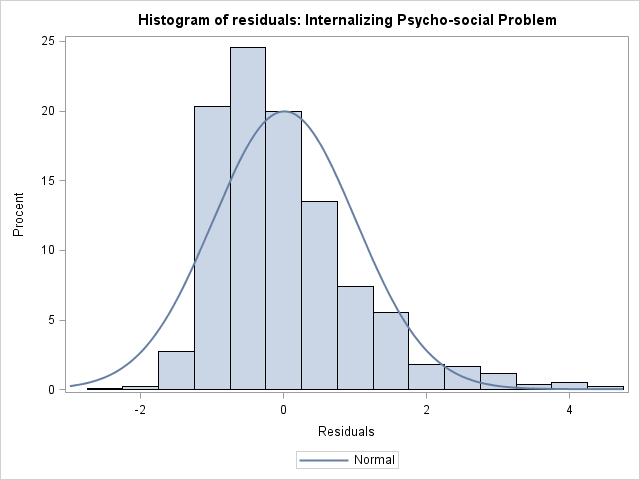

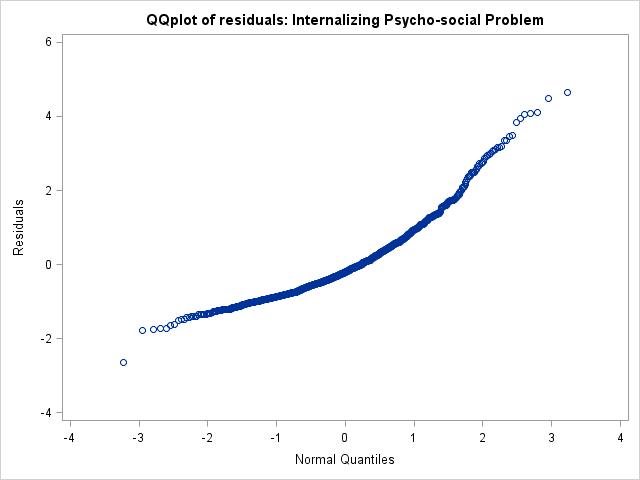
**
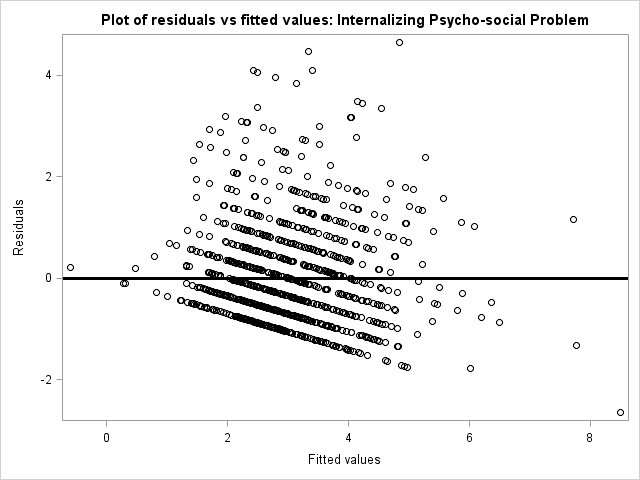
**

# 7. Distribution of SES and other Covariates by ECEC-start-age in West and East Germany

**Table S4.1 Distribution of SES by ECEC-start-age in West and East Germany**

|  | West Germany (n=621) | | | East Germany (n=401) | | |
| --- | --- | --- | --- | --- | --- | --- |
|  | Low SES (%) | Middle SES (%) | High SES (%) | Low SES (%) | Middle SES (%) | High SES (%) |
| Below age 1 | 3.2 | 4.1 | 14.4 | 11.7 | 21.2 | 37.3 |
| Age 1-2 | 1.2 | 4.7 | 12.6 | 47.0 | 47.8 | 36.2 |
| Age 2-3 | 39.1 | 44.8 | 41.9 | 35.5 | 26.1 | 21.9 |
| Age 3+ and before schooling | 27.6 | 30.9 | 21.6 | 5.9 | 3.6 | 3.9 |
| Only cared in Family before schooling | 28.9 | 15.6 | 9.6 | 0 | 1.4 | 0.7 |
| Total | 47 | 365 | 203 | 46 | 243 | 111 |
| Chi-Square | **P<0.0001** | | | P=0.151 | | |

The weight factor for baseline data was applied in the frequency and statistical tests. The number of missing values for West Germany was 6, and for East Germany was 1.

**Table S4.2 Distribution of Family Structure by ECEC-start-age in West and East Germany**

|  | West Germany (n=621) | | East Germany (n=401) | |
| --- | --- | --- | --- | --- |
|  | Natural parents in a joint household (%) | Other Family Structure (%) | Natural parents in a joint household (%) | Other Family Structure (%) |
| Below age 1 | 6.2 | 16.0 | 24.5 | 23.4 |
| Age 1-2 | 6.1 | 11.7 | 47.3 | 29.7 |
| Age 2-3 | 43.2 | 44.3 | 23.7 | 38.9 |
| Age 3+ and before schooling | 27.9 | 25.3 | 3.3 | 8.0 |
| Only cared in Family before schooling | 16.7 | 2.7 | 1.2 | 0 |
| Total | 572 | 45 | 338 | 59 |
| Chi-Square | **P=0.007** | | P=0.179 | |

“other family structure” refers to the family structures except for “Natural parents in a joint household”.

The weight factor for baseline data was applied in the frequency and statistical tests. The number of missing values for West Germany was 4, and for East Germany was 4.

**Table S4.3 Distribution of Migration Background by ECEC-start-age in West and East Germany**

|  | West Germany (n=621) | | East Germany (n=401) | |
| --- | --- | --- | --- | --- |
|  | Non-migrant (%) | Migrant (%) | Non-migrant (%) | Migrant (%) |
| Below age 1 | 7.7 | 2.1 | 25.9 | 2.1 |
| Age 1-2 | 7.1 | 3.3 | 44.6 | 42.8 |
| Age 2-3 | 42.4 | 47.7 | 24.6 | 48.9 |
| Age 3+ and before schooling | 27.5 | 29.6 | 4.3 | 0 |
| Only cared in Family before schooling | 15.3 | 17.3 | 0.7 | 6.1 |
| Total | 540 | 78 | 387 | 10 |
| Chi-Square | P=0.080 | | P=0.155 | |

The weight factor for baseline data was applied in the frequency and statistical tests. The number of missing values for West Germany was 3, and for East Germany was 4.

**Table S4.4 Distribution of Mother’s Employment Status by ECEC-start-age in West and East Germany**

|  | West Germany (n=621) | | | East Germany (n=401) | | |
| --- | --- | --- | --- | --- | --- | --- |
|  | Full-time employed (%) | Part-time employed (%) | Unemployed (%) | Full-time employed (%) | Part-time employed (%) | Unemployed (%) |
| Below age 1 | 23.9 | 8.0 | 3.4 | 36.1 | 23.0 | 11.6 |
| Age 1-2 | 9.1 | 9.0 | 4.3 | 47.0 | 51.7 | 33.6 |
| Age 2-3 | 35.6 | 43.4 | 43.9 | 14.3 | 21.9 | 45.0 |
| Age 3+ and before schooling | 22.1 | 28.0 | 28.2 | 2.1 | 2.9 | 7.5 |
| Only cared in Family before schooling | 9.2 | 11.6 | 20.1 | 0.5 | 0.5 | 2.3 |
| Total | 53 | 253 | 304 | 141 | 129 | 128 |
| Chi-Square | **P=0.005** | | | **P=0.004** | | |

The weight factor for baseline data was applied in the frequency and statistical tests. The number of missing values for West Germany was 11, and for East Germany was 3.

**Table S4.5 Distribution of Age of Mother at Birth by ECEC-start-age in West and East Germany**

|  | West Germany (n=621) | | | | East Germany (n=401) | | | |
| --- | --- | --- | --- | --- | --- | --- | --- | --- |
|  | Till 24 years old | 25-29 years old | 30-34 years old | 35+ years old | Till 24 years old | 25-29 years old | 30-34 years old | 35+ years old |
| Below age 1 | 6.1 | 5.7 | 4.0 | 13.9 | 26.1 | 26.2 | 24.2 | 17.9 |
| Age 1-2 | 4.8 | 8.2 | 5.5 | 7.5 | 43.1 | 45.4 | 46.3 | 40.7 |
| Age 2-3 | 48.1 | 44.1 | 42.3 | 39.6 | 24.8 | 23.3 | 26.1 | 34.0 |
| Age 3+ and before schooling | 25.4 | 26.8 | 29.8 | 27.7 | 3.9 | 5.1 | 2.9 | 3.7 |
| Only cared in Family before schooling | 15.5 | 15.1 | 18.4 | 11.3 | 2.1 | 0 | 0.5 | 3.8 |
| Total | 68 | 136 | 247 | 158 | 66 | 143 | 127 | 54 |
| Chi-square | P=0.128 | | | | P=0.311 | | | |

The weight factor for baseline data was applied in the frequency and statistical tests. The number of missing values for West Germany was 12, and for East Germany was 11.

Note: The p-values keep in the same direction even after excluding the rows with 0 cells in East Germany for these Tables (S4.1-S4.5).

# 8. Table to support Figures 2 and 3 in the main paper

**Table S5.1. Mean values and 95% CI of psycho-social problems in adolescence by ECEC-start-age group**

|  | ECEC-start-age groups |  | SDQ Total Score | |  | Externalizing Psycho-social Problems | |  | Internalizing Psycho-social Problems | |
| --- | --- | --- | --- | --- | --- | --- | --- | --- | --- | --- |
|  |  | n | Mean (95%CI) |  |  | Mean (95%CI) |  |  | Mean (95%CI) |  |
| Germany  (n=1022) | Below age 1 | 144 | 8.10 (6.93, 9.28) |  |  | 4.99 (4.23, 5.74) |  |  | 3.12 (2.47, 3.77) |  |
|  | Age 1-2 | 214 | 7.72 (6.78, 8.67) |  |  | 4.69 (4.05, 5.32) |  |  | 3.04 (2.61, 3.46) |  |
|  | Age 2-3 | 366 | 6.81 (6.23, 7.40) |  |  | 3.96 (3.53, 4.38) |  |  | 2.85 (2.52, 3.18) |  |
|  | Age 3+ and before schooling | 205 | 7.71 (7.00, 8.42) |  |  | 4.21 (3.79, 4.63) |  |  | 3.50 (3.08, 3.91) |  |
|  | Only cared in Family before schooling | 93 | 8.15 (6.60, 9.71) |  |  | 5.08 (4.09, 6.08) |  |  | 3.07 (2.29, 3.85) |  |

**Table S5.2. Mean values and 95% CI of psycho-social problems in adolescence by ECEC-start-age group and region**

|  | ECEC-start-age groups |  | SDQ Total Score | |  | Externalizing Psycho-social Problems | |  | Internalizing Psycho-social Problems | |
| --- | --- | --- | --- | --- | --- | --- | --- | --- | --- | --- |
|  |  | n | Mean (95%CI) |  |  | Mean (95%CI) |  |  | Mean (95%CI) |  |
| West Germany  (n=621) | Below age 1 | 45 | 9.17 (7.34, 11.00) |  |  | 5.60 (4.43, 6.77) |  |  | 3.57 (2.50, 4.65) |  |
|  | Age 1-2 | 41 | 8.16 (5.81, 10.51) |  |  | 5.04 (3.43, 6.64) |  |  | 3.12 (2.15, 4.10) |  |
|  | Age 2-3 | 261 | 6.67 (6.03, 7.32) |  |  | 3.94 (3.46, 4.42) |  |  | 2.74 (2.39, 3.08) |  |
|  | Age 3+ and before schooling | 185 | 7.63 (6.91, 8.35) |  |  | 4.18 (3.75, 4.61) |  |  | 3.45 (3.03, 3.87) |  |
|  | Only cared in Family before schooling | 89 | 8.15 (6.57, 9.73) |  |  | 5.09 (4.08, 6.10) |  |  | 3.07 (2.27, 3.86) |  |
| East Germany  (n=401) | Below age 1 | 99 | 6.70 (5.69, 7.70) |  |  | 4.18 (3.55, 4.81) |  |  | 2.51 (1.94, 3.09) |  |
|  | Age 1-2 | 173 | 7.47 (6.75, 8.18) |  |  | 4.48 (4.04, 4.93) |  |  | 2.99 (2.58, 3.39) |  |
|  | Age 2-3 | 105 | 7.72 (6.47, 8.98) |  |  | 4.09 (3.35, 4.82) |  |  | 3.64 (2.61, 4.66) |  |
|  | Age 3+ and before schooling | 20 | 9.81 (5.34, 14.27) |  |  | 5.14 (2.89, 7.39) |  |  | 4.67 (2.22, 7.11) |  |
|  | Only cared in Family before schooling | 4 | 8.10 (5.36, 10.85) |  |  | 4.92 (-0.11, 9.95) |  |  | 3.19 (0.49, 5.88) |  |

Reference

1. Lampert T, Hoebel J, Kuntz B, Müters S, Kroll L. Socioeconomic status and subjective social status measurement in KiGGS Wave 2 2018; 3(1).

2. Hölling H, Kurth B-M, Rothenberger A, Becker A, Schlack R. Assessing psychopathological problems of children and adolescents from 3 to 17 years in a nationwide representative sample: results of the German health interview and examination survey for children and adolescents (KiGGS). European Child & Adolescent Psychiatry 2008; 17 Suppl 1:34–41.

3. Schneewind KA, Beckmann M, Hecht-Jackl A. Das FK-Testsystem. Inst. für Psychologie, Persönlichkeitspsychologie und Psychodiagnostik, Universität München; 1985.

4. Abubakari A, Kynast-Wolf G, Jahn A. Prevalence of abnormal birth weight and related factors in Northern region, Ghana. BMC Pregnancy Childbirth 2015; 15:335.

5. Kromeyer-Hauschild K, Wabitsch M, Kunze D, Geller F, Geiß HC, Hesse V et al. Perzentile für den Body-mass-Index für das Kindes- und Jugendalter unter Heranziehung verschiedener deutscher Stichproben. Monatsschr Kinderheilkd 2001; 149(8):807–18.

6. Büchner C, Spiess CK. Die Dauer vorschulischer Betreuungs-und Bildungserfahrungen: Ergebnisse auf der Basis von Paneldaten. SOEPpapers on Multidisciplinary Panel Data ResearchResearch, No. 10, Deutsches Institut für Wirtschaftsforschung; 2007.

7. Klipker K, Baumgarten F, Göbel K, Lampert T, Hölling H. Psychische Auffälligkeiten bei Kindern und Jugendlichen in Deutschland – Querschnittergebnisse aus KiGGS Welle 2 und Trends. Journal of Health Monitoring 2018; 3(3).

8. Hinshaw SP. Process, mechanism, and explanation related to externalizing behavior in developmental psychopathology. Journal of abnormal child psychology 2002; 30(5):431–46.

9. Dohmen D, Karrmann E, Bayreuther T. Entwicklung frühkindlicher Bildungsbedarfe in Berlin: Vom Platzmangel zu Bildungschancen: Projekt in Kooperation mit: Kita-Stimme.berlin; 2021.

10. Presch K. Wer bekommt einen Kitaplatz? Das sind die Auswahlkriterien der Bönener Kitas; 2020 [cited 2023 Jul 10]. Available from: URL: https://www.wa.de/lokales/boenen/bekommt-einen-kitaplatz-boenener-kitas-legen-ihre-kriterien-selbst-fest-13529812.html.

11. Fukuya Y, Fujiwara T, Isumi A, Doi S, Ochi M. Association of Birth Order With Mental Health Problems, Self-Esteem, Resilience, and Happiness Among Children: Results From A-CHILD Study. Front Psychiatry 2021; 12:638088.

12. NICHD Early Child Care Research Network. Nonmaternal care and family factors in early development: An overview of the NICHD Study of Early Child Care. Journal of Applied Developmental Psychology 2001; 22(5):457–92.

13. Qin Y, Wan X, Qu S, Chen G. Family Cohesion and School Belonging in Preadolescence: Examining the Mediating Role of Security and Achievement Goals. SHS Web of Conferences 2015; 19:2004.

14. Damota MD. The Effect of Divorce on Families's Life. JCSD 2019; (46).

15. Gennaro S. Leave and employment in families of preterm low birthweight infants. Image J Nurs Sch 1996; 28(3):193–8.

16. Mathewson KJ, Chow CHT, Dobson KG, Pope EI, Schmidt LA, van Lieshout RJ. Mental health of extremely low birth weight survivors: A systematic review and meta-analysis. Psychol Bull 2017; 143(4):347–83.

17. Storz MA. The COVID-19 pandemic: an unprecedented tragedy in the battle against childhood obesity. Clin Exp Pediatr 2020; 63(12):477–82.

18. Sagar R, Gupta T. Psychological Aspects of Obesity in Children and Adolescents. Indian J Pediatr 2018; 85(7):554–9.

19. Khoshnood B, Wall S, Lee K. Risk of low birth weight associated with advanced maternal age among four ethnic groups in the United States. Matern Child Health J 2005; 9(1):3–9.

20. Bundeszentrale für politische Bildung. Kinder in Tagesbetreuung; 2021 [cited 2022 Nov 10]. Available from: URL: https://www.bpb.de/nachschlagen/zahlen-und-fakten/soziale-situation-in-deutschland/61615/kinder-in-tagesbetreuung.

21. Henry-Huthmacher C. Kinderbetreuung in Deutschland – Ein Übeblick: Konrad-Adenauer-Stiftung; 2005 [cited 2022 Nov 10]. Available from: URL: https://www.kas.de/c/document_library/get_file?uuid=8d691d49-dd5b-b43c-0852-1f9ba4052aeb&groupId=252038.

22. Röbenack S. Der lange Weg zur Einheit – Die Entwicklung der Arbeitslosigkeit in Ost- und Westdeutschland: Bundeszentrale für politische Bildung; 2020 [cited 2022 Nov 11]. Available from: URL: https://www.bpb.de/themen/deutsche-einheit/lange-wege-der-deutschen-einheit/47242/der-lange-weg-zur-einheit-die-entwicklung-der-arbeitslosigkeit-in-ost-und-westdeutschland/.

23. Bohn V, Rathmann K, Richter M. Psychosoziale Gesundheit bei Kindern und Jugendlichen in Nordrhein-Westfalen: Die Bedeutung von Alter, Geschlecht und Schultyp. Gesundheitswesen 2010; 72(5):293–300.
